# Immunogenicity and Safety of Co-administration of Adjuvanted Respiratory Syncytial Virus Prefusion F Protein Vaccine With 20-Valent Pneumococcal Conjugate Vaccine in Adults Aged ≥60 Years: A Randomized, Non-inferiority Trial

**DOI:** 10.1093/cid/ciag250

**Published:** 2026-04-13

**Authors:** Isabel Leroux-Roels, Muriel Lins, Ronald Surowitz, Antoinette Pragalos, Paulia Kwo Ton Ge Jua, Dominique Wauters, Amulya Jayadev, Jacqueline Elaine Akite, Archana M Jastorff, Vicky Cárdenas, Marie Van der Wielen, RSV-019 study group, Abram Burgher, Adeyemi Gbohunmi, Agnieszka Jurek-Urbanowska, Bruce Rankin, Carlos Brotons Cuixart, Ewa Krecipro Nizinska, Fernando Cereto Castro, Jay Sandberg, Jean Benoit Martinot, Joseph Soufer, Katarzyna Wawrzyniec-Lis, Kathleen Jones, Klaas Vercruysse, Lina Perez Breva, Luc Capiau, Magda Dabrowska, Manuchehr Darani, Miguel Trevino, Patrick Dennis, Patrick Soentjens, Przemyslaw Borowy, Rachel Presti, Rica Santiago, Salma Saiger, Sander Gothard, Shital Upadhyay, Tatyana Miroshnikova, William Nixon, Madhavi Ampajwala

**Affiliations:** Center for Vaccinology, Ghent University and Ghent University Hospital, Ghent, Belgium; AZ Sint-Maarten, Mechelen, Department of Pulmonology, Belgium; Jupiter Medical Center, Jupiter, Florida, USA; Christ Hospital, Cincinnati, Ohio, USA; GSK, Wavre, Belgium; GSK, Wavre, Belgium; GSK, Bangalore, India; GSK, Wavre, Belgium; GSK, Wavre, Belgium; GSK, Rockville, Maryland, USA; GSK, Wavre, Belgium

**Keywords:** immunogenicity, older adults, pneumococcal conjugate vaccine, respiratory syncytial virus, safety

## Abstract

**Background:**

Respiratory syncytial virus (RSV) illness and pneumococcal disease present high disease burden and health risks to older adults. Vaccination against these 2 illnesses is a key strategy to reduce this burden, and co-administration of the 2 vaccines could enhance vaccine uptake. This study evaluated the immunogenicity, safety, and reactogenicity of co-administration of adjuvanted RSV prefusion F protein vaccine (adjuvanted RSVPreF3) with 20-valent pneumococcal conjugate vaccine (PCV20) in adults aged ≥60 years.

**Methods:**

This phase III, open-label, randomized, non-inferiority trial was conducted across 38 centers in Belgium, Poland, Spain, and the United States. Participants (N = 1112) were randomized 1:1 to receive adjuvanted RSVPreF3 vaccine and PCV20 vaccine either concomitantly (Co-Ad group) or 1 month apart (Control group). The primary objectives were to assess immunogenicity as measured by RSV-A and RSV-B neutralizing titers and opsonophagocytic titers for the PCV20 serotypes. Safety and reactogenicity were assessed as secondary objectives.

**Results:**

All primary immunogenicity objectives were met. The upper limit of the 95% confidence interval of the geometric mean titer ratios for RSV-A, RSV-B, and all 20 PCV20 serotypes was within the pre-defined non-inferiority margins. Safety profiles were similar across both groups, with most adverse events being mild to moderate in severity and of short duration. Serious adverse events and potential immune-mediated diseases were rare and unrelated to vaccination.

**Conclusions:**

Co-administration of adjuvanted RSVPreF3 vaccine and PCV20 vaccine in older adults is immunologically non-inferior to their sequential administration, with an acceptable safety profile. These findings support co-administration as a strategy to enhance vaccine uptake.

**Clinical Trials Registration:**

NCT05879107.

## Teaser

Co-administration of adjuvanted RSVPreF3 vaccine and PCV20 vaccine in adults aged ≥60 years was similarly immunogenic as administration of the 2 vaccines 1 month apart, with a similar safety profile. These results support simplified vaccination schedules to improve vaccination coverage.

Respiratory syncytial virus (RSV) is a highly contagious virus that infects the respiratory tract, mostly affecting infants and young children, older adults, and individuals with comorbidities [1, 2]. In adults ≥ 60 years of age and in immunocompromised individuals, RSV infection presents a significant burden, although it is often underrecognized as a cause of illness due to lack of routine testing [3–5]. The first symptoms of RSV infection are nonspecific and often associated with other viral infections, such as influenza. Due to delayed diagnosis, disease progression can be severe, with an increased likelihood of lower respiratory tract involvement [6]. In older adults in high-income countries, an estimated 470 000 hospitalizations and 33 000 in-hospital deaths due to RSV infection were reported in 2019 [3].

Vaccination can help reduce the burden of RSV in older adults [7, 8]. The AS01_E_-adjuvanted RSV prefusion F protein-based vaccine (adjuvanted RSVPreF3, *Arexvy*, GSK) is efficacious against RSV-associated lower respiratory tract disease over at least 3 seasons in older adults and has a clinically acceptable safety profile [9]. In the United States, a single dose of RSV vaccine is recommended for all adults aged ≥75 years and for adults aged 50–74 years who are at increased risk of severe RSV disease [10]. In Europe, vaccination guidance varies by country, with recommendations tailored to specific risk groups and/or age groups [11]. For example, RSV vaccination may be recommended for all adults aged ≥60 years or specifically for those with certain diseases or comorbidities.

*Streptococcus pneumoniae* causes a variety of illnesses, ranging from relatively mild (eg, bronchitis, sinusitis) to potentially life-threatening (eg, septicemia, pneumonia, meningitis) [12]. Vaccination against *S. pneumoniae* is a cornerstone prevention strategy in most countries. In the United States, vaccination with a multivalent pneumococcal vaccine is recommended for all adults aged ≥50 years, as well as for younger adults at increased risk of pneumococcal disease [13]. In Europe, most countries recommend multivalent vaccines for adults ≥ 65 years and for all adults at increased risk of pneumococcal disease [14].

The co-administration of vaccines is a beneficial strategy to increase vaccination coverage and reduce the burden of vaccination on patients and on healthcare systems. Adjuvanted RSVPreF3 vaccine can be co-administered with influenza vaccines and herpes zoster vaccines in older adults without clinically significant alterations to the safety profile or immune responses [15–17]. Pneumococcal vaccines can be co-administered with influenza or with SARS-CoV-2 vaccines in adults [18]. Using a non-inferiority design, we investigated the immunogenicity, safety, and reactogenicity of adjuvanted RSVPreF3 vaccine and 20-valent pneumococcal conjugate vaccine (PCV20) when co-administered compared to when the 2 vaccines are administered 1 month apart. A plain language summary of this study is provided in Figure 1.

**Figure 1.**
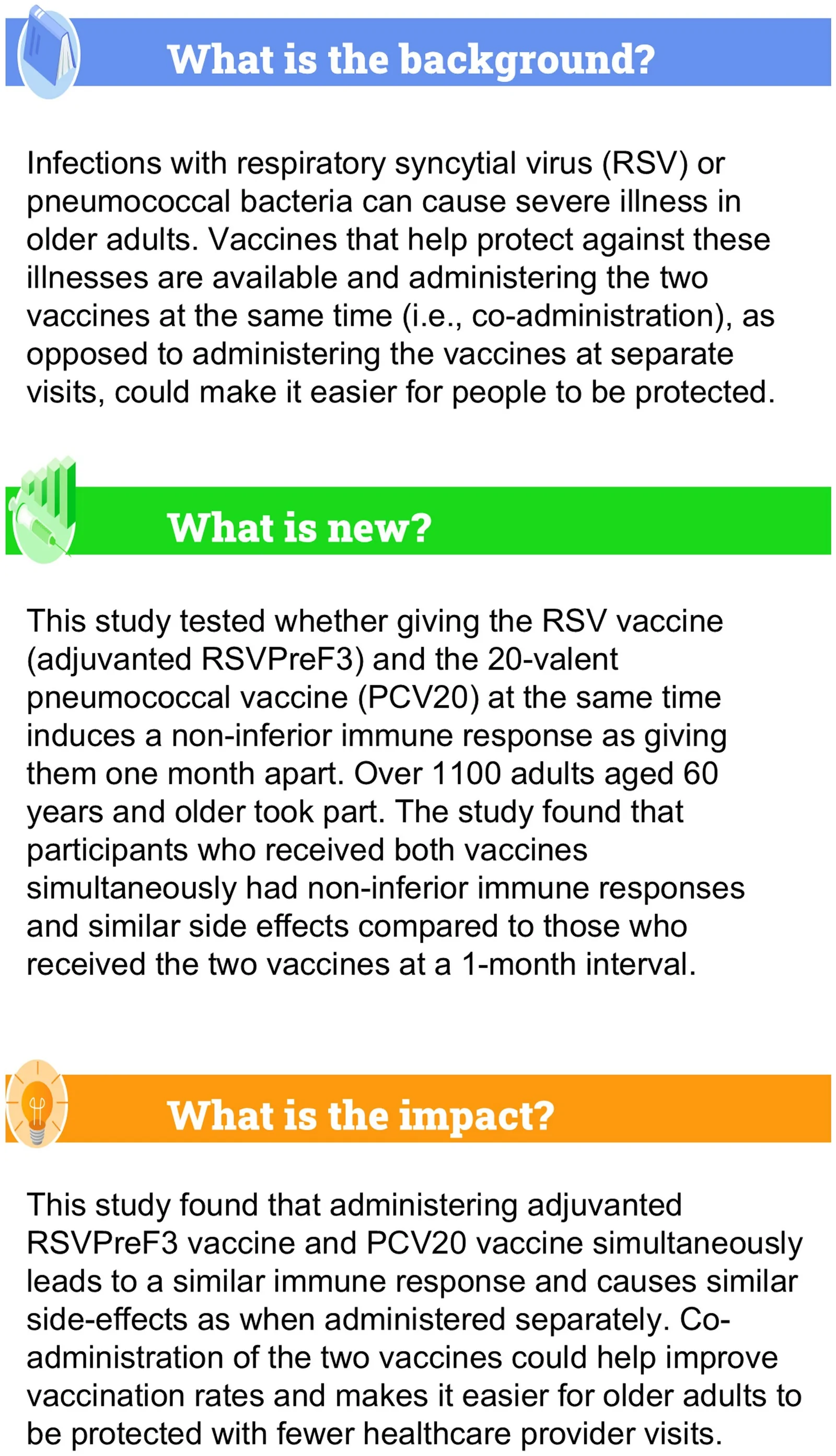
Plain language summary.

## METHODS

### Ethics

This study was designed and executed in accordance with the ethical principles contained in applicable international guidelines and applicable laws and regulations. Study-related documents were approved by institutional or national ethical review boards. Prior to enrollment into the study, informed consent was obtained from each participant.

The study is registered on EudraCT (2022-501988-40-00) and ClinicalTrials.gov (NCT05879107). Full study protocol and statistical analysis plan are available at gsk-studyregister.com (study 219276).

### Study Design and Procedures

This phase III open-label, randomized, controlled, non-inferiority study was performed at 38 study centers in 4 countries (Belgium, Poland, Spain, and the United States) between May 2023 and May 2024.

Participants were randomly assigned to one of 2 parallel intervention groups (1:1 ratio using a centralized internet-based system). A minimization procedure accounting for age category (60–69, 70–79, and ≥80 years of age) and study center was used, with both factors having equal weight. Participants in the Co-Ad group received 1 intramuscular dose of adjuvanted RSVPreF3 vaccine in the non-dominant arm and 1 intramuscular dose of PCV20 vaccine (*Prevnar 20/Prevenar 20*, Pfizer) in the dominant arm, co-administered on Day 1. Participants in the Control group received 1 intramuscular dose of PCV20 vaccine in the non-dominant arm on Day 1, followed 1 month later by 1 intramuscular dose of adjuvanted RSVPreF3 vaccine in the non-dominant arm (Figure 2). Neither participants nor investigators were blinded to the study treatment. The central laboratory in charge of sample testing was blinded to treatment group.

**Figure 2.**
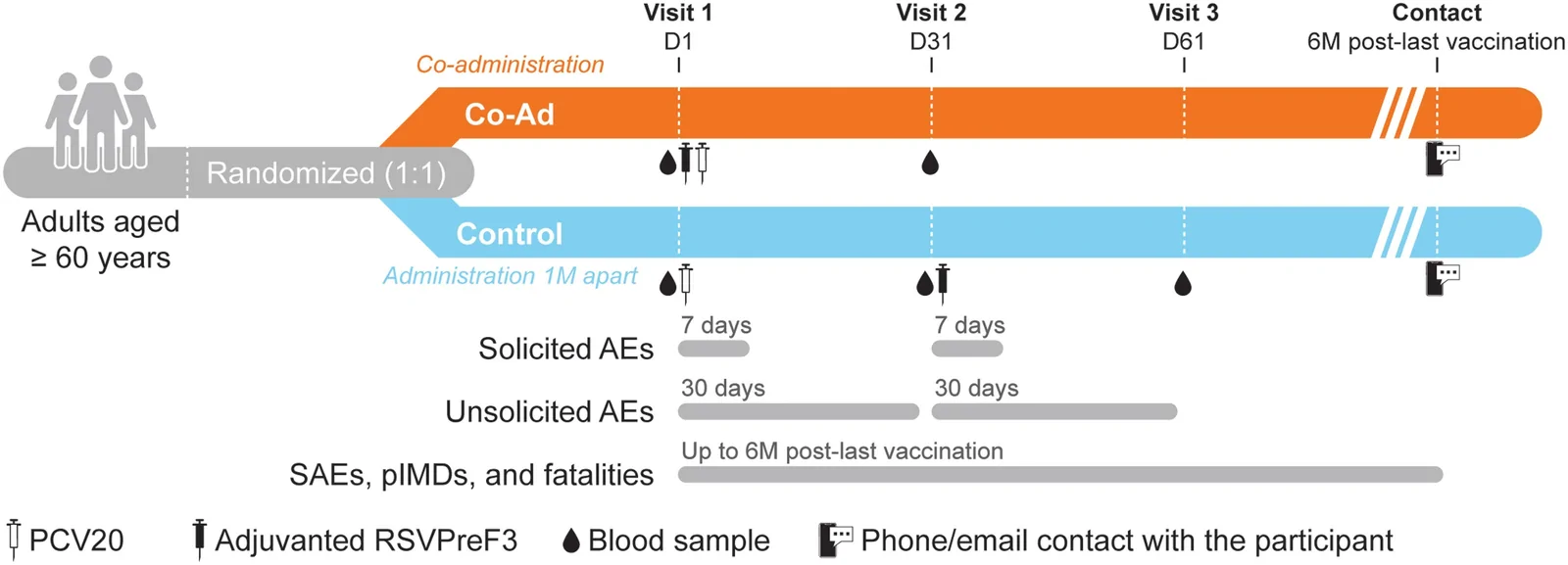
Study design and procedures. Co-Ad, participants who received a single dose of adjuvanted RSVPreF3 in the non-dominant arm and a single dose of PCV20 in the dominant arm on Day 1; Control, participants who received a single dose of PCV20 on Day 1 and a single dose of adjuvanted RSVPreF3 on Day 31, both administered (preferably) in the non-dominant arm. Abbreviations: AEs, adverse events; D, day; M, month; PCV20, 20-valent pneumococcal conjugate vaccine; pIMDs, potential immune-mediated diseases; RSV, respiratory syncytial virus; SAEs, serious adverse events.

On Day 1, demographic data, medical history, and vaccination history were collected. Blood samples were collected from all participants pre-vaccination on Day 1 and 30 days post-vaccination (Day 31 for participants in the Co-Ad group and Days 31 and 61 for participants in the Control group). Immune responses were assessed by measuring RSV-A and RSV-B neutralizing titers and PCV20 serotype-specific *S. pneumoniae* opsonophagocytic titers. Titers below the assay cutoff were assigned half the assay cutoff value; titers above the upper limit of quantification were replaced by the upper limit of quantification. Immunogenicity analyses were performed on log_10_-transformed data.

Safety data were collected at the time of study visits or by using electronic diary cards. Solicited administration-site and systemic adverse events (AEs) were collected for 7 days post-vaccination. If a symptom persisted beyond the initial 7-day post-vaccination period, the collection period could be extended to 30 days post-vaccination. The intensity of all solicited AEs was graded (1 to 3) by the participant (see Supplementary Table 1) and confirmed by the investigator for measurable events (eg, fever and diameter of injection site reactions). Unsolicited AEs were collected for 30 days post-vaccination (Day 1 to Day 30 for participants in both groups, plus Day 31 to Day 60 for participants in the Control group), and serious AEs (SAEs) and potential immune-mediated diseases (pIMDs; Supplementary Table 2) were collected until study conclusion (6 months after the last vaccine dose, ie, Month 6 for participants in the Co-Ad group, Month 7 for participants in the Control group). Intensity of AEs and causal relation to the study vaccination were assessed by the investigator.

### Study Population

Medically stable adults, ≥60 years of age at the time of the first study vaccination, without any confirmed or suspected immunosuppressive or immunodeficient conditions were eligible. The main exclusion criteria were previous or planned vaccination with a PCV, receipt of a pneumococcal polysaccharide vaccine in the previous five years or during the study, or previous receipt of an RSV vaccine. Detailed inclusion and exclusion criteria are listed in the Supplementary Data.

### Objectives

The study had 2 co-primary objectives: to demonstrate that co-administration of adjuvanted RSVPreF3 vaccine with PCV20 vaccine is non-inferior to the sequential administration of these vaccines (adjuvanted RSVPreF3 given 1 month after PCV20), based on geometric mean RSV-A neutralizing titers (GMT; in 60% effective dose [ED60]) 1 month post-vaccination, and to demonstrate that co-administration of PCV20 vaccine with adjuvanted RSVPreF3 vaccine is non-inferior to their sequential administration, based on opsonophagocytic titers for each PCV20 serotype 1 month post-vaccination. If these 2 co-primary objectives were met, non-inferiority of adjuvanted RSVPreF3 in terms of RSV-B neutralizing titers was assessed.

Secondary objectives of the study were to evaluate the mean geometric increase (MGI) of RSV-A and RSV-B neutralizing titers 1 month after the adjuvanted RSVPreF3 dose compared to baseline titers, as well as the assessment of the safety and reactogenicity of adjuvanted RSVPreF3 vaccine and PCV20 vaccine when co-administered or administered 1 month apart. Full details of study objectives and endpoints are provided in the Supplementary Table 3.

### Statistical Analysis

A sample size of 1090 participants was targeted to obtain at least 980 evaluable participants (490 in each treatment group), resulting in 93.7% power to meet the primary objectives, with a nominal type I error of 2.5%.

All immunogenicity assessments were based on the per-protocol set, ie, the set of all participants who complied with all protocol-specified requirements and who had pre- and post-dose immunogenicity results available. For non-inferiority testing, GMT ratios (Control group divided by Co-Ad group) were first calculated for RSV-A neutralizing titers and for opsonophagocytic titers for each of the 20 PCV20 serotypes. Non-inferiority was achieved if the upper limit of the 2-sided 95% confidence interval (CI) of the GMT ratio was ≤1.5 for RSV-A neutralizing titers and ≤2 for each PCV20 serotype. Upon success of this first sequence, non-inferiority was similarly tested for RSV-B neutralizing titers (upper limit of the 2-sided 95% CI of the GMT ratio ≤ 1.5). The non-inferiority margin of 1.5 for RSV was based on feedback from the US Food & Drug Administration Center for Biologics Evaluation and Research; the margin of 2 for PCV20 serotypes was based on the criteria used during the development of PCV20 [19]. Additionally, the primary immunogenicity analyses were repeated on the exposed set, ie, the set of all enrolled participants who received at least 1 study intervention.

Secondary immunogenicity objectives were evaluated by calculating the MGI for within-participant ratios of post- versus pre-dose RSV-A/RSV-B neutralizing titers. Geometric mean fold rise (GMFR) of opsonophagocytic titer post- versus pre-dose was calculated for each of the PCV20 serotypes, as well as the proportion of participants showing a ≥4-fold increase post- versus pre-dose for both RSV-A/RSV-B neutralizing titers and opsonophagocytic titers. Safety and reactogenicity evaluations were analyzed descriptively in the exposed set, ie, all participants who received at least 1 dose of any study vaccine. Considering an imbalance in the number of concomitant non-study vaccinations between the 2 study groups, a post hoc analysis of the safety data was performed on the exposed set excluding participants who received a concomitant non-study vaccination within 30 days of a study vaccination and reported an unsolicited AE.

## RESULTS

### Study Participants

Overall, 1112 participants were randomized. Of these, 1110 (99.8%) received at least 1 study vaccine (exposed set; 553 participants in the Co-Ad group, 557 participants in the Control group) and 1070 participants (96.4%) completed the study. The main reasons for not completing the study were “loss to follow-up” (21 participants, 1.9%) and “withdrawal by participant due to reasons not related to AEs” (12 participants, 1.1%). The per-protocol set for RSV immunogenicity consisted of 513 participants in the Co-Ad group and 383 participants in the Control group, while the per-protocol set for PCV20 immunogenicity consisted of 514 participants in the Co-Ad group and 497 participants in the Control group (Figure 3).

**Figure 3.**
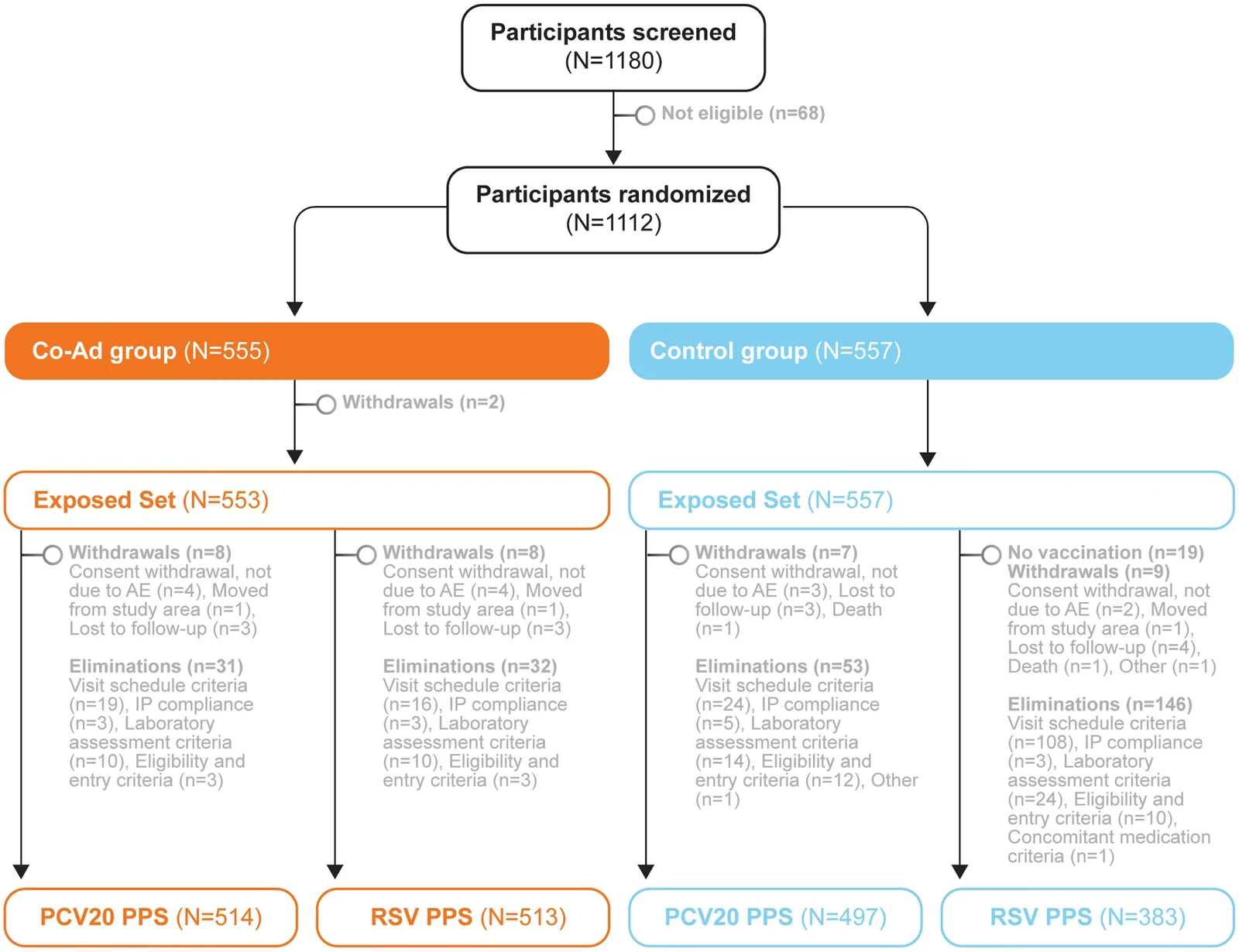
Participant disposition flowchart. Co-Ad, participants who received a single dose of adjuvanted RSVPreF3 and a single dose of PCV20 on Day 1; Control, participants who received a single dose of PCV20 on Day 1 and a single dose of adjuvanted RSVPreF3 on Day 31. Participants could have multiple reasons for elimination. “Visit schedule criteria” refers to an out-of-window study visit (allowed window for Visit 2: 30–42 days post-Visit 1). “Laboratory assessment criteria” refers to missed pre- or post-dose immunogenicity samples (eg, sample not taken, shipping issue, sample viability issue). Abbreviations: AE, adverse event; IP, investigational product; N, number of participants in each group or set; n, number of participants in each category; PCV20, 20-valent pneumococcal conjugate vaccine; PPS, per-protocol set; RSV, respiratory syncytial virus.

Participant demographics were well balanced between the groups. The overall median age was 69 years at the time of first vaccination, with 51.4% of the participants in the 60–69 years category. Most participants were White (86.8%), not Hispanic/Latino (95.3%), residing in the United States (66.0%), and female (56.8%) (Table 1). Overall, 13 participants (2.4%) in the Co-Ad group and 56 participants (10.1%) in the Control group received a vaccine other than the study vaccines within 30 days of a study vaccination visit. Most of these were SARS-CoV-2 vaccines (9 participants [1.6%] in the Co-Ad group, 41 participants [7.4%] in the Control group).

**Table 1.** Participant Demographics (Exposed Set)

Category	Co-Ad (N = 553)	Control (N = 557)	Total (N = 1110)
Age (y) at first dose			
Mean (SD)	69.6 (7.28)	69.6 (7.41)	69.6 (7.35)
Median (Min–Max)	69.0 (60–93)	69.0 (60–92)	69.0 (60–93)
Age category (y), n (%)			
60–69	283 (51.2)	287 (51.5)	570 (51.4)
70–79	201 (36.3)	201 (36.1)	402 (36.2)
≥80	69 (12.5)	69 (12.4)	138 (12.4)
≥65	383 (69.3)	368 (66.1)	751 (67.7)
Sex, n (%)			
Male	237 (42.9)	242 (43.4)	479 (43.2)
Female	316 (57.1)	315 (56.6)	631 (56.8)
Country, n (%)			
Belgium	100 (18.1)	103 (18.5)	203 (18.3)
Poland	76 (13.7)	76 (13.6)	152 (13.7)
Spain	11 (2.0)	11 (2.0)	22 (2.0)
US	366 (66.2)	367 (65.9)	733 (66.0)
Race, n (%)			
Black or African American	64 (11.6)	54 (9.7)	118 (10.6)
White	476 (86.1)	488 (87.6)	964 (86.8)
Other^a^	13 (2.4)	15 (2.7)	28 (2.5)
Ethnicity, n (%)			
Hispanic or Latino	28 (5.1)	23 (4.1)	51 (4.6)
Not Hispanic or Latino	524 (94.8)	534 (95.9)	1058 (95.3)
Missing	1 (0.2)	0	1 (0.1)

Co-Ad, participants who received a single dose of adjuvanted RSVPreF3 and a single dose of PCV20 on Day 1; Control, participants who received a single dose of PCV20 on Day 1 and a single dose of adjuvanted RSVPreF3 on Day 31. Abbreviations: Max, maximum; Min, minimum; N, number of participants in each group; n (%), number and percentage of participants in each category; PCV20, 20-valent pneumococcal conjugate vaccine; RSV, respiratory syncytial virus; SD, standard deviation; US, United States.

^a^Includes American Indian or Alaska Native, Asian, Native Hawaiian or Other Pacific Islanders, multiple, and other races.

### Non-inferiority of Co-administration of Adjuvanted RSVPreF3 and PCV20

One month after receipt of the adjuvanted RSVPreF3 vaccine dose, the upper limit of the 2-sided 95% CI of the adjusted GMT ratio of the Control group (at Day 61) over the Co-Ad group (at Day 31) was 1.20 for RSV-A neutralizing titers and 1.13 for RSV-B neutralizing titers, thus demonstrating non-inferiority (pre-defined criterion ≤ 1.5) (Figure 4). The additional analysis, performed on the exposed set, showed similar results (Supplementary Table 4).

**Figure 4.**
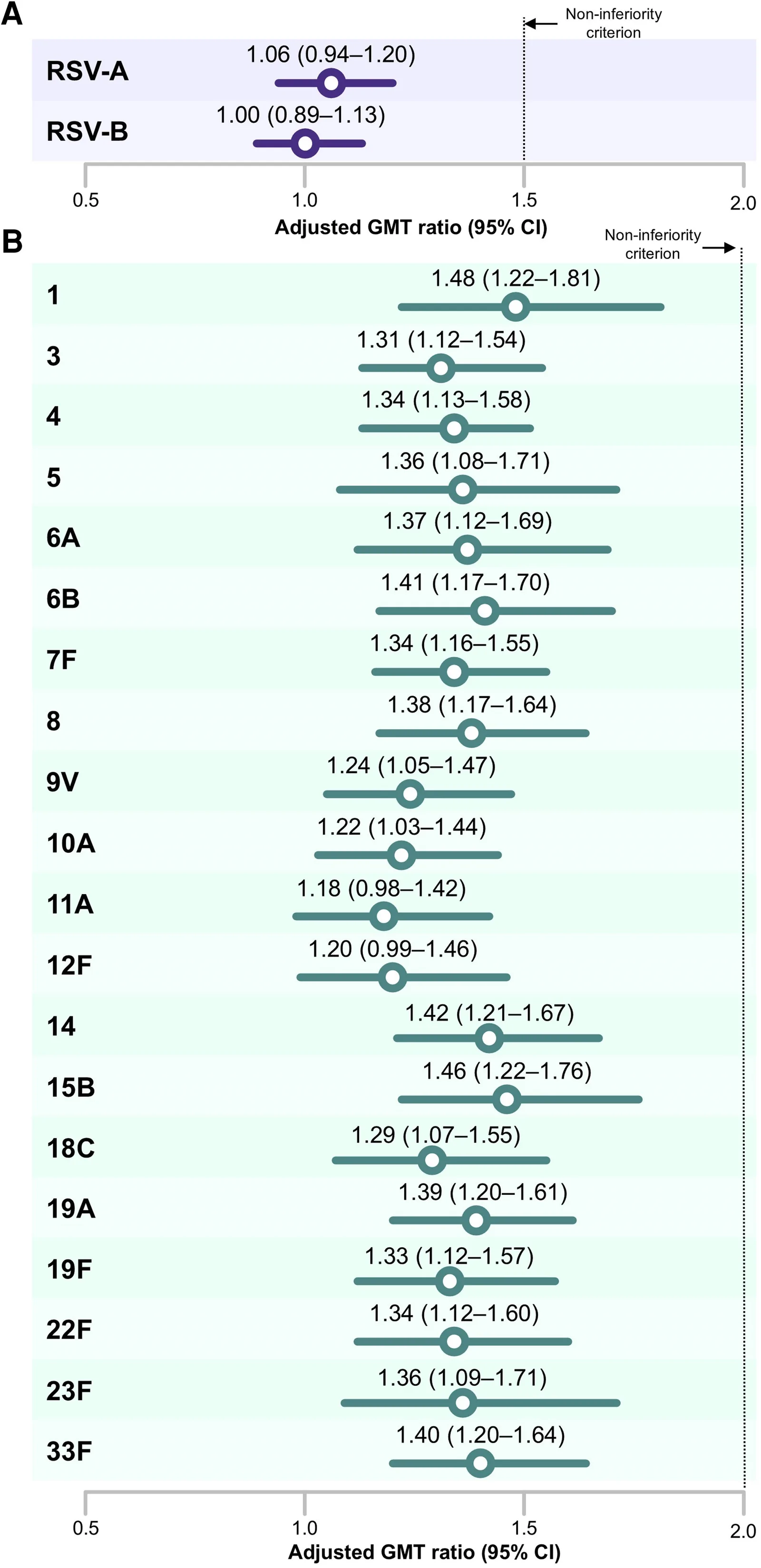
Adjusted geometric mean titer ratios of the Control over the Co-Ad group 1 month post-vaccination for PCV20 serotypes (opsonophagocytic assay) and RSV-A and RSV-B (neutralization assay) in the per-protocol sets. Co-Ad, participants who received a single dose of adjuvanted RSVPreF3 and a single dose of PCV20 on Day 1; Control, participants who received a single dose of PCV20 on Day 1 and a single dose of adjuvanted RSVPreF3 on Day 31. Abbreviations: CI, confidence interval; GMT, geometric mean titer; N, number of participants with available results; PCV20, 20-valent pneumococcal conjugate vaccine; RSV-A/B, respiratory syncytial virus subtype A/B.

The non-inferiority criterion for the PCV20 immune response was also achieved as the upper limit of the 2-sided 95% CI for the ratio of the adjusted GMTs of the Control group over the Co-Ad group (both at Day 31) was ≤2 for each of the 20 PCV20 serotypes (Figure 4).

### Immune Response to Adjuvanted RSVPreF3 and PCV20

RSV-A neutralizing titers increased from pre-vaccination to 1 month after vaccination in both groups (1026.4–8533.5 ED60 in the Co-Ad group, 960.8–8880.9 ED60 in the Control group), resulting in an MGI of 8.3 in the Co-Ad group and 9.3 in the Control group (Figure 5). In the Co-Ad group, 75.4% (95% CI: 71.4–79.1) of participants showed a ≥4-fold increase in RSV-A neutralizing titers 1 month after vaccination versus 79.3% (95% CI: 74.9–83.3) of participants in the Control group. Similarly, RSV-B neutralizing titers increased from 1008.8 to 9393.5 ED60 from pre-vaccination to 1 month after vaccination in the Co-Ad group versus 1053.6 to 9760.0 ED60 in the Control group, with MGIs of 9.4 in the Co-Ad group and 9.1 in the Control group (Figure 5). A ≥4-fold increase in RSV-B neutralizing titers was observed in 79.8% (95% CI Co-Ad group: 76.1–83.2; Control group: 75.5–83.8) of participants in both groups.

**Figure 5.**
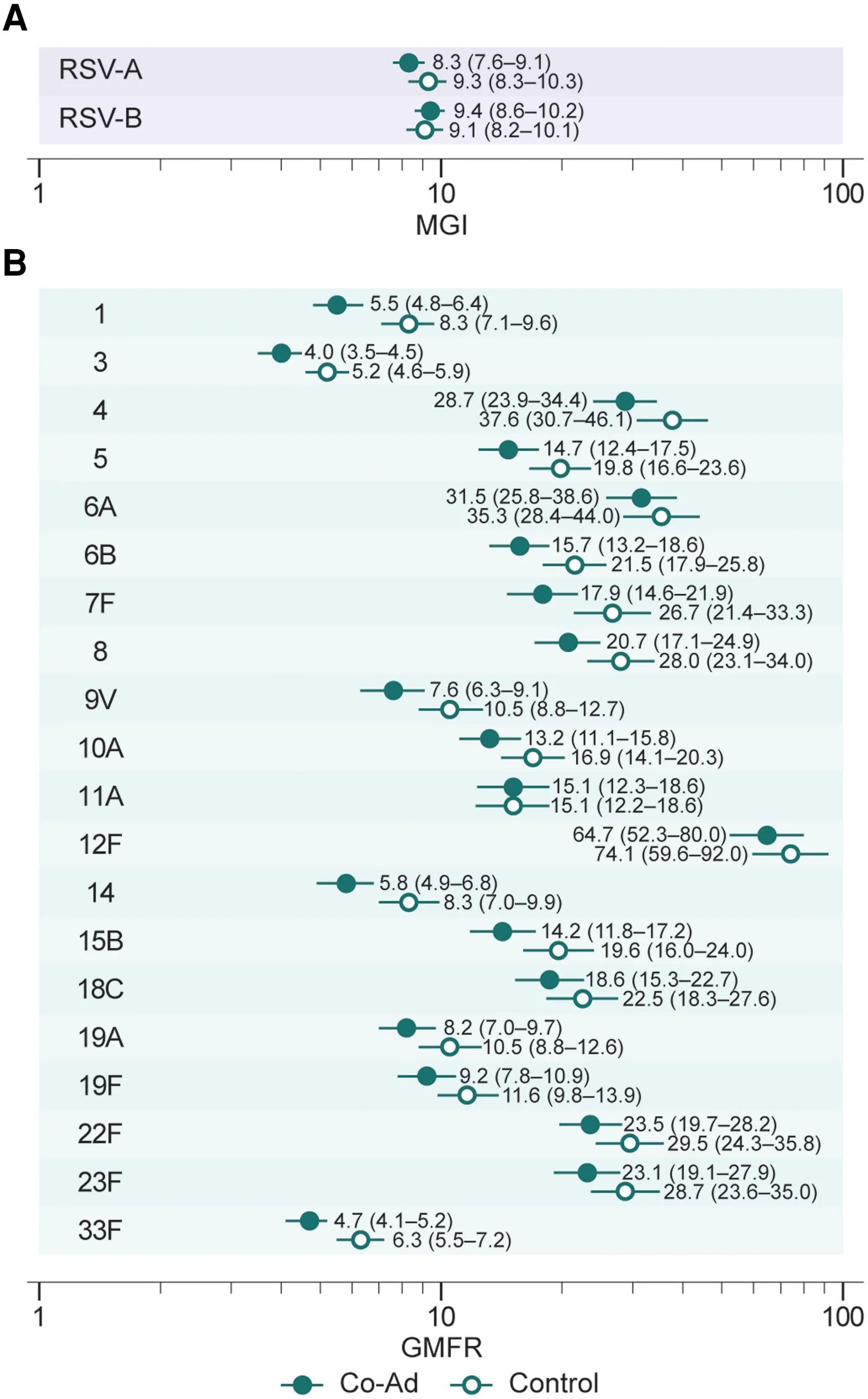
MGIs for RSV neutralizing titers and GMFRs for pneumococcal opsonophagocytic titers from pre-vaccination to 1 month post-vaccination (per-protocol sets for RSV and PCV20). Co-Ad, participants who received a single dose of adjuvanted RSVPreF3 and a single dose of PCV20 on Day 1; Control, participants who received a single dose of PCV20 on Day 1 and a single dose of adjuvanted RSVPreF3 on Day 31. Abbreviations: GMFR, geometric mean fold rise; MGI, mean geometric increase; PCV20, 20-valent pneumococcal conjugate vaccine; RSV-A/B, respiratory syncytial virus subtype A/B.

The GMFR of opsonophagocytic titers ranged from 4.0- to 64.7-fold across all PCV20 serotypes in the Co-Ad group and from 5.2- to 74.1-fold in the Control group 1 month after vaccination compared to pre-vaccination (Figure 5). Opsonophagocytic titers increased ≥4-fold in 44.4% to 79.5% of participants in the Co-Ad group and in 54.5% to 83.4% of participants in the Control group.

### Safety and Reactogenicity Results

Within 7 days of any dose, at least 1 solicited administration-site AE was reported by 348 (64.7%) participants in the Co-Ad group and 325 (58.9%) participants in the Control group (Figure 6). The most frequently reported event in both groups was pain at the administration site, reported by 336 (62.5%) participants in the Co-Ad group and 312 (56.5%) participants in the Control group. Grade 3 solicited administration-site AEs were reported by 13 (2.4%) participants in the Co-Ad group and 6 (1.1%) participants in the Control group. Across both groups, the median duration of each solicited administration-site event was 3 days or less. Frequencies of solicited administration-site events per participant and per dose are shown in Supplementary Table 5.

**Figure 6.**
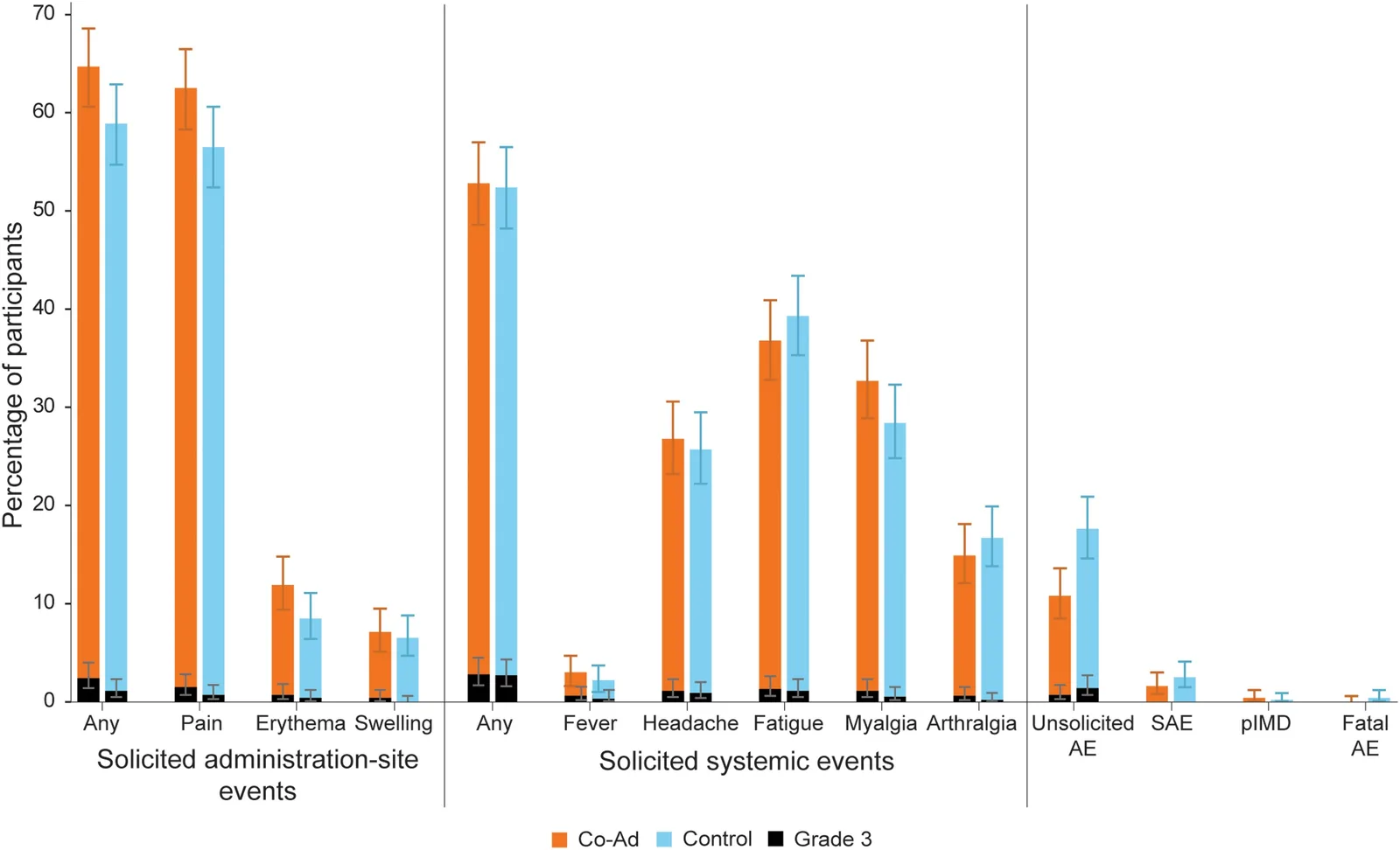
Adverse events (overall per participant, exposed set). Co-Ad, participants who received a single dose of adjuvanted RSVPreF3 and a single dose of PCV20 on Day 1; Control, participants who received a single dose of PCV20 on Day 1 and a single dose of adjuvanted RSVPreF3 on Day 31. Abbreviations: AE, adverse event; PCV20, 20-valent pneumococcal conjugate vaccine; pIMD, potential immune-mediated disease; SAE, serious adverse event.

At least 1 solicited systemic AE within 7 days of any dose was reported by 284 (52.8%) participants in the Co-Ad group and 289 (52.4%) participants in the Control group (Figure 6). The most frequently reported solicited systemic event was fatigue, reported by 198 (36.8%) participants in the Co-Ad group and 217 (39.3%) participants in the Control group. In both groups, 15 participants (2.8% and 2.7%) reported at least 1 grade 3 solicited systemic AE. The median duration of each event across both groups was 2 days or less.

In the Co-Ad group, within 30 days of receiving any dose, 60 participants (10.8%) reported a total of 86 unsolicited AEs, 4 of which (reported by 4 participants [0.7%]) were grade 3 events. The investigator assessed 21 events in 17 (3.1%) participants as related to the study vaccines. In the Control group, 98 (17.6%) participants reported 147 unsolicited AEs, of which 9 (reported by 8 participants [1.4%]) were grade 3 events. Fifteen events reported by 11 (2.0%) participants were considered related to the study vaccines by the investigator.

Up to study end, 9 participants (1.6%) in the Co-Ad group and 14 participants (2.5%) in the Control group reported at least 1 SAE. Three participants (2 [0.4%] in the Co-Ad and 1 [0.2%] in the Control group) reported a pIMD. The investigators determined that no SAE or pIMD was related to the study interventions. Two fatal SAEs (0.4%) were reported, both occurring in the Control group. One participant in the 60–69 years of age group died 76 days after receiving adjuvanted RSVPreF3 (Day 107); one participant in the ≥80 years of age group died on Day 18, ie, 17 days after receiving PCV20. The cause of death was unknown for both participants but assessed as unrelated to the study interventions by the investigator.

The post hoc analysis, excluding participants who received a concomitant non-study vaccination, showed similar results in terms of reported incidence of unsolicited AEs and SAEs (Supplementary Table 6).

## DISCUSSION

All primary objectives for immunogenicity were met. The upper limit of the 2-sided 95% CI of the adjusted GMT ratio of the Control group over the Co-Ad group 1 month after vaccination was ≤1.5 for RSV-A and RSV-B neutralizing titers and ≤2 for opsonophagocytic titers against each of the 20 PCV20 serotypes. Therefore, co-administering adjuvanted RSVPreF3 with PCV20 is immunologically non-inferior to administering both vaccines 1 month apart.

The MGIs and percentages of participants with ≥4-fold increase in RSV-A/RSV-B neutralizing titers were comparable when adjuvanted RSVPreF3 was co-administered with PCV20 versus when administered 1 month after PCV20. The opsonophagocytic titers for all PCV20 serotypes increased post-vaccination across groups and serotypes. A ≥4-fold increase was noted in 44% to 83% of participants, which was comparable between both groups and indicates that co-administration of both vaccines had no substantial effect on the PCV20 response. In the absence of a validated correlate of protection for PCVs in adults, small differences in the opsonophagocytic titers may be acceptable, but the clinical relevance is uncertain, especially for low-response serotypes, such as serotype 3.

The incidence of most solicited AEs was comparable between the 2 study groups; most were of mild to moderate intensity, with low frequencies of grade 3 (severe) events, and a short median duration. A greater number of unsolicited AEs were reported when the study vaccines were given sequentially compared to when they were co-administered. This is likely attributable to the additional safety reporting period for the Control group (ie, 2 vaccination visits) versus the single reporting period for the Co-Ad group (ie, one vaccination visit). A post hoc analysis did not indicate that the imbalance was attributable to the larger number of concomitant non-study vaccine administered in the Control group. Furthermore, at each individual visit, the percentages of participants experiencing unsolicited AEs were similar (data not shown). The frequencies of reported SAEs and pIMDs were similar across the study groups and none were considered related to study interventions. Overall, we found no clinically meaningful differences in the safety profile of adjuvanted RSVPreF3 and PCV20 when co-administered versus given 1 month apart.

Despite well-balanced demographics between study groups, a limitation of our study is that we only included participants from high-income countries, with a small proportion of participants above 80 years of age. The limited diversity of the enrolled participants might limit the generalizability of our findings; however, in the pivotal phase 3 efficacy study of the adjuvanted RSVPreF3 vaccine, no significant differences between races or ethnicities in terms of efficacy or safety were reported [9]. The open-label design and relatively large number of eliminations from the per-protocol set for adjuvanted RSVPreF3 immunogenicity in the Control group were also limitations. Many of the eliminations in the Control group in the per-protocol set occurred due to an out-of-window Visit 2 caused by participant scheduling conflicts (travel, illness, etc.). This observation illustrates a challenge of sequential administration of vaccines and supports the added value of vaccine co-administration. Although some numerical differences in terms of adjusted GMTs were found when considering the exposed set versus the per-protocol set, these differences do not change the conclusions of our primary analyses.

Based on our results and general principles of co-administration of protein-based vaccines, it is reasonable to infer that adjuvanted RSVPreF3 vaccine can be co-administered with any PCV. Similar to PCV20, other currently available PCVs recommended for adult immunization include serotypes covalently linked to CRM197 [20]. CRM197 is a widely used carrier protein for conjugate vaccines, and CRM197-conjugated vaccines are routinely co-administered with other vaccines across the world. An increase in the number of pneumococcal serotypes and/or higher dosage of CRM197 is unlikely to interfere with the immunogenicity or safety profiles of either vaccine upon co-administration with adjuvanted RSVPreF3 [21–23].

Co-administration of adult vaccines can safely and effectively improve vaccine uptake [24]. The favorable safety and immunogenicity profiles observed in this study support the use of co-administration strategies in routine clinical practice, potentially improving coverage without compromising immune responses or tolerability.

## CONCLUSION

We demonstrated that co-administration of adjuvanted RSVPreF3 vaccine with PCV20 vaccine is immunologically non-inferior to administration of the 2 vaccines 1 month apart, with an acceptable reactogenicity and safety profile. These findings could aid in increasing RSV vaccination coverage, thereby decreasing the burden for patients as well as for healthcare systems.

## Supplementary Material

ciag250_Supplementary_Data

## Supplementary Data

Supplementary materials are available at *Clinical Infectious Diseases* online. Consisting of data provided by the authors to benefit the reader, the posted materials are not copyedited and are the sole responsibility of the authors, so questions or comments should be addressed to the corresponding author.
