# Supplementary material for: Immunogenicity and Safety of Co-administration of Adjuvanted Respiratory Syncytial Virus Prefusion F Protein Vaccine With 20-Valent Pneumococcal Conjugate Vaccine in Adults Aged ≥60 Years: A Randomized, Non-inferiority Trial
Source: Clin Infect Dis. 2026 Apr 13;83(3):e589–97. doi: 10.1093/cid/ciag250 (PMC13644879; doi:10.1093/cid/ciag250)
Supplement: Supplementary file 1 [file ciag250_supplementary_data.docx]

**Immunogenicity and safety of co-administration of adjuvanted respiratory syncytial virus prefusion F protein vaccine with 20-valent pneumococcal conjugate vaccine in adults aged ≥60 years: a randomized, non-inferiority trial**

Isabel Leroux-Roels, Muriel Lins, Ronald Surowitz, Antoinette Pragalos, Paulia Kwo Ton Ge Jua, Dominique Wauters, Amulya Jayadev, Jacqueline Elaine Akite, Archana M Jastorff, Vicky Cárdenas, Marie Van der Wielen, on behalf of the RSV-019 study group

**Supplementary Material**

**Table of contents:**

Detailed inclusion and exclusion criteria	2

Supplementary Table 1. Intensity scales for solicited events	5

Supplementary Table 2. List of potential immune-mediated diseases (pIMDs)	7

Supplementary Table 3. Study objectives and endpoints	14

Supplementary Table 4. Additional analysis of primary RSV immunogenicity endpoints	15

Supplementary Table 5. Solicited administration site events within 7 days following each dose and overall (exposed set)	16

Supplementary Table 6. Summary of unsolicited AEs, SAEs and pIMDs (exposed set, without and with post‑hoc adjustment for receipt of concomitant non‑study vaccination)	18

Detailed inclusion and exclusion criteria

All participants must satisfy **all** the following criteria at study entry:

- A male or female ≥60 years of age at the time of the first study intervention administration.
- Participants who, in the opinion of the investigator, can and will comply with the requirements of the protocol (e.g., completion of the eDiary, return for follow-up visits, ability to access and utilize a phone or other electronic communications).
  *Note: In case of physical incapacity that would preclude the self-completion of the eDiary, either site staff can assist the participant (for activities performed during site visits) or the participant may assign a caregiver* to assist him/her with this activity (for activities performed at home). However, at no time will the site staff or caregiver* evaluate the participant’s health status while answering diaries or make decisions on behalf of the participant. *A ‘caregiver’ is a person who has a continuous caring role for a participant or may be a person having substantial periods of contact with a participant and/or is engaged in his/her daily health care (e.g., a relative of the participant including family members or friends).*
- Written or witnessed informed consent obtained from the participant prior to any study-specific procedure being performed.
- Participants living in the general community or in an assisted-living facility that provides minimal assistance, such that the participant is primarily responsible for self-care and activities of daily living.
- Participants who are medically stable in the opinion of the investigator at the time of first study intervention administration. Participants with chronic stable medical conditions with or without specific treatment, such as diabetes mellitus, hypertension, or cardiac disease, are allowed to participate in this study if considered by the investigator as medically stable.

The following criteria should be checked at the time of study entry. The potential participant **may not** be included in the study if **any** exclusion criterion applies:

*Medical conditions*

- Any confirmed or suspected immunosuppressive or immunodeficient condition resulting from disease (e.g., current malignancy, human immunodeficiency virus) or immunosuppressive/cytotoxic therapy (e.g., medication used during cancer chemotherapy, organ transplantation or to treat autoimmune disorders), based on medical history and physical examination (no laboratory testing required).
- History of any reaction or hypersensitivity (e.g., anaphylaxis) likely to be exacerbated by the study interventions, in particular any history of severe allergic reaction to any vaccine containing diphtheria toxoid (for example, diphtheria-tetanus-pertussis), or 23-valent pneumococcal polysaccharide vaccine.
- Participants considered by the investigator as suffering from serious or unstable chronic illness.
- Any history of dementia or any medical condition that moderately or severely impairs cognition.
  *Note: If deemed necessary for clinical evaluation, the investigator can use tools such as Mini-Mental State Examination, Mini-Cog or Montreal Cognitive Assessment to determine cognition levels of the participant.*
- Recurrent or uncontrolled neurological disorders or seizures. Participants with medically controlled chronic neurological diseases can be enrolled in the study as per investigator assessment, provided that their condition will allow them to comply with the requirements of the protocol (e.g., completion of the eDiary, attend regular phone calls/study site visits).
- Significant underlying illness that in the opinion of the investigator would be expected to prevent completion of the study (e.g., life-threatening disease likely to limit survival up to the end of the study).
- Any medical condition that in the judgment of the investigator would make intramuscular injection unsafe.

*Prior and concomitant therapy*

- History of previous vaccination with any licensed or investigational pneumococcal conjugate vaccine, or planned receipt through study participation.
- History of previous vaccination with any licensed or investigational pneumococcal polysaccharide vaccine in the last five years from enrollment, or planned receipt through study participation.
- Previous vaccination with any licensed or investigational RSV vaccine.
- Use of any investigational or non-registered product (drug, vaccine or medical device) other than the study interventions during the period beginning 30 days before the first dose of study interventions and ending 30 days after the last study intervention administration, or their planned use during the study period.
- Planned or actual administration of a vaccine not foreseen by the study protocol in the period starting 30 days before the first study intervention administration and ending 30 days after the last study intervention administration.
  - Planned or actual administration of adjuvanted quadrivalent influenza vaccine or live influenza vaccine not foreseen by the study protocol in the period starting 30 days before the first study intervention administration and ending 30 days after the last study intervention administration.
    *Note: In case an emergency mass vaccination for an unforeseen public health threat (e.g., a pandemic) is recommended and/or organized by the public health authorities outside the routine immunization program, the time period described above can be reduced if necessary for that vaccine, provided it is used according to the local governmental recommendations and that the Sponsor or designee is notified accordingly.*
- Administration of long-acting immune-modifying drugs during the period starting 180 days before the administration of the first dose of study interventions or planned administration at any time during the study period (e.g., infliximab).
- Administration of immunoglobulins and/or any blood products or plasma derivatives during the period starting 90 days before the administration of the first dose of study interventions or planned administration during the study period.
- Chronic administration (defined as more than 14 consecutive days in total) of immunosuppressants or other immune-modifying drugs during the period starting 90 days prior to the first study intervention dose or planned administration during the study period. For corticosteroids, this will mean prednisone ≥20 mg/day, or equivalent. Inhaled and topical steroids are allowed.

*Prior / Concurrent clinical study experience*

- Concurrently participating in another clinical study, at any time during the study period, in which the participant has been or will be exposed to an investigational or a non-investigational vaccine/product (drug or invasive medical device).
  *Note: European Economic Community (EEC) directive 93/42/EEC defines an invasive medical device as ‘A device which, in whole or in part, penetrates inside the body, either through a body orifice or through the surface of the body.’*

*Other exclusions*

- History of chronic alcohol consumption and/or drug abuse as deemed by the investigator to render the potential participant unable/unlikely to provide accurate safety reports or comply with study procedures.
- Bedridden participants.
- Planned move during the study conduct that prohibits participation until study end.
- Participation of any study personnel or their immediate dependents, family or household members.

Supplementary Table 1. Intensity scales for solicited events

| **Event** | **Intensity grade** | **Parameter** |
| --- | --- | --- |
| Pain at administration site | 0 | None |
|  | 1 | Mild: Any pain neither interfering with nor preventing normal everyday activities |
|  | 2 | Moderate: Painful when limb is moved and interferes with everyday activities |
|  | 3 | Severe: Significant pain at rest. Prevents normal everyday activities |
| Erythema at administration site* | Greatest surface diameter in mm | |
| Swelling at administration site* | Greatest surface diameter in mm | |
| Temperature^*^ | Temperature in °C or °F | |
| Headache | 0 | None |
|  | 1 | Mild: Headache that is easily tolerated |
|  | 2 | Moderate: Headache that interferes with normal activity |
|  | 3 | Severe: Headache that prevents normal activity |
| Fatigue | 0 | None |
|  | 1 | Mild: Fatigue that is easily tolerated |
|  | 2 | Moderate: Fatigue that interferes with normal activity |
|  | 3 | Severe: Fatigue that prevents normal activity |
| Myalgia | 0 | None |
|  | 1 | Mild: Myalgia that is easily tolerated |
|  | 2 | Moderate: Myalgia that interferes with normal activity |
|  | 3 | Severe: Myalgia that prevents normal activity |
| Arthralgia | 0 | None |
|  | 1 | Mild: Arthralgia that is easily tolerated |
|  | 2 | Moderate: Arthralgia that interferes with normal activity |
|  | 3 | Severe: Arthralgia that prevents normal activity |

* The maximum intensity of local administration-site erythema/swelling and fever will be scored by the investigator as follows:

| **Intensity Grade** | **Erythema/Swelling** | **Fever** |
| --- | --- | --- |
| 0 | ≤20 mm | <38.0°C (100.4°F) |
| 1 | >20 – ≤50 mm | ≥38.0°C (100.4°F) – ≤38.5°C (101.3°F) |
| 2 | >50 – ≤100 mm | >38.5°C (101.3°F) – ≤39.0°C (102.2°F) |
| 3 | >100 mm | >39.0°C (102.2°F) |

Supplementary Table 2. List of potential immune-mediated diseases (pIMDs)

| Medical Concept | Additional Notes | |
| --- | --- | --- |
| Blood disorders and coagulopathies |  | |
| Antiphospholipid syndrome |  | |
| Autoimmune aplastic anemia |  | |
| Autoimmune hemolytic anemia | Includes warm antibody hemolytic anemia and cold antibody hemolytic anemia | |
| Autoimmune lymphoproliferative syndrome (ALPS) |  | |
| Autoimmune neutropenia |  | |
| Autoimmune pancytopenia |  | |
| Autoimmune thrombocytopenia | Frequently used related terms include: “autoimmune thrombocytopenic purpura”, “idiopathic thrombocytopenic purpura (ITP)”, “idiopathic immune thrombocytopenia”, “primary immune thrombocytopenia”. | |
| Evans syndrome |  | |
| Pernicious anemia |  | |
| Thrombosis with thrombocytopenia syndrome (TTS) |  | |
| Thrombotic thrombocytopenic purpura | Also known as “Moschcowitz-syndrome” or “microangiopathic hemolytic anemia” | |
| Cardio-pulmonary inflammatory disorders |  | |
| Idiopathic Myocarditis/Pericarditis | Including but not limited to:   - Autoimmune / Immune-mediated myocarditis - Autoimmune / Immune-mediated pericarditis - Giant cell myocarditis | |
| Idiopathic pulmonary fibrosis | Including but not limited to:   - Idiopathic interstitial pneumonia (frequently used related terms include “Interstitial lung disease”, “Pulmonary fibrosis”, “Immune-mediated pneumonitis”) - Pleuroparenchymal fibroelastosis (PPFE) | |
| Pulmonary alveolar proteinosis (PAP) | Frequently used related terms include: “pulmonary alveolar lipoproteinosis”, “phospholipidosis” | |
| Endocrine disorders |  | |
| Addison’s disease |  | |
| Autoimmune / Immune-mediated thyroiditis | Including but not limited to:   - Hashimoto thyroiditis (autoimmune hypothyroidism, lymphocytic thyroiditis) - Atrophic thyroiditis - Silent thyroiditis - Thyrotoxicosis | |
| Autoimmune diseases of the testis and ovary | Includes autoimmune oophoritis, autoimmune ovarian failure and autoimmune orchitis | |
| Autoimmune hyperlipidemia |  | |
| Autoimmune hypophysitis |  | |
| Diabetes mellitus type I |  | |
| Grave's or Basedow’s disease | Includes Marine Lenhart syndrome and Graves' ophthalmopathy, also known as thyroid eye disease (TED) or endocrine ophthalmopathy |  |
| Insulin autoimmune syndrome |  |  |
| Polyglandular autoimmune syndrome | Includes Polyglandular autoimmune syndrome type I, II and III |  |
| Eye disorders |  |  |
| Ocular Autoimmune / Immune-mediated disorders | Including but not limited to:   - Acute macular neuroretinopathy (also known as acute macular outer retinopathy) - Autoimmune / Immune-mediated retinopathy - Autoimmune / Immune-mediated uveitis, including idiopathic uveitis and sympathetic ophthalmia - Cogan's syndrome: an oculo-audiovestibular disease - Ocular pemphigoid - Ulcerative keratitis - Vogt-Koyanagi-Harada disease |  |
| Gastrointestinal disorders |  |  |
| Autoimmune / Immune-mediated pancreatitis |  |  |
| Celiac disease |  |  |
| Inflammatory Bowel disease | Including but not limited to:   - Crohn’s disease - Microscopic colitis - Terminal ileitis - Ulcerative colitis - Ulcerative proctitis |  |
| Hepatobiliary disorders |  |  |
| Autoimmune cholangitis |  |  |
| Autoimmune hepatitis |  |  |
| Primary biliary cirrhosis |  |  |
| Primary sclerosing cholangitis |  |  |
| Musculoskeletal and connective tissue disorders |  |  |
| Gout | Includes gouty arthritis |  |
| Idiopathic inflammatory myopathies | Including but not limited to:   - Dermatomyositis - Inclusion body myositis - Immune-mediated necrotizing myopathy - Polymyositis |  |
| Mixed connective tissue disorder |  |  |
| Polymyalgia rheumatica (PMR) |  |  |
| Psoriatic arthritis (PsA) |  |  |
| Relapsing polychondritis |  |  |
| Rheumatoid arthritis | Including but not limited to:   - Rheumatoid arthritis associated conditions - Juvenile idiopathic arthritis - Palindromic rheumatism - Still's disease - Felty’s syndrome |  |
| Sjögren’s syndrome |  |  |
| Spondyloarthritis | Including but not limited to:   - Ankylosing spondylitis - Juvenile spondyloarthritis - Keratoderma blenorrhagica - Psoriatic spondylitis - Reactive Arthritis (Reiter's Syndrome) - Undifferentiated spondyloarthritis |  |
| Systemic lupus Erythematosus | Includes Lupus associated conditions (e.g., Cutaneous lupus erythematosus, Lupus nephritis, etc.) or complications such as shrinking lung syndrome (SLS) |  |
| Systemic Scleroderma (systemic sclerosis) | Includes Reynolds syndrome (RS), systemic sclerosis with diffuse scleroderma and systemic sclerosis with limited scleroderma (also known as CREST syndrome) |  |
| Neuroinflammatory/neuromuscular disorders |  |  |
| Acute disseminated encephalomyelitis (ADEM) and other inflammatory demyelinating variants | Includes the following:   - Acute necrotizing myelitis - Bickerstaff's brainstem encephalitis - Disseminated necrotizing leukoencephalopathy (also known as Weston-Hurst syndrome, acute hemorrhagic leuko-encephalitis, or acute necrotizing hemorrhagic encephalomyelitis) - Myelin oligodendrocyte glycoprotein antibody-associated disease - Neuromyelitis optica (also known as Devic’s disease) - Noninfective encephalitis / encephalomyelitis / myelitis - Postimmunization encephalomyelitis |  |
| Guillain-Barré syndrome (GBS) | Includes variants such as Miller Fisher syndrome and the acute motor and sensory axonal neuropathy (AMSAN) |  |
| Idiopathic cranial nerve palsies/paresis and inflammations (neuritis) | Including but not limited to:   - Cranial nerve neuritis (e.g., Optic neuritis) - Idiopathic nerve palsies/paresis (e.g., Bell’s palsy) - Melkersson-Rosenthal syndrome - Multiple cranial nerve palsies/paresis |  |
| Multiple sclerosis (MS) | Includes the following:   - Clinically isolated syndrome (CIS) - Malignant MS (the Marburg type of MS) - Primary-progressive MS (PPMS) - Radiologically isolated syndrome (RIS) - Relapsing-remitting MS (RRMS) - Secondary-progressive MS (SPMS) - Uhthoff's phenomenon |  |
| Myasthenia gravis | Includes ocular myasthenia and Lambert-Eaton myasthenic syndrome |  |
| Narcolepsy | Includes narcolepsy with or without presence of unambiguous cataplexy |  |
| Peripheral inflammatory demyelinating neuropathies and plexopathies | Including but not limited to:   - Acute Brachial Radiculitis (also known as Parsonage-Turner Syndrome or neuralgic amyotrophy) - Antibody-mediated demyelinating neuropathy - Chronic idiopathic axonal polyneuropathy (CIAP) - Chronic Inflammatory Demyelinating Polyradiculoneuropathy (CIDP), including atypical CIDP variants (e.g., multifocal acquired demyelinating sensory and motor neuropathy also known as Lewis-Sumner syndrome) - Multifocal motor neuropathy (MMN) |  |
| Transverse myelitis (TM) | Includes acute partial transverse myelitis (APTM) and acute complete transverse myelitis (ACTM) |  |
| Renal disorders |  |  |
| Autoimmune / immune-mediated glomerulonephritis | Including but not limited to:   - Immunoglobulin A (IgA) nephropathy - Immunoglobulin M (IgM) nephropathy - C1q nephropathy - Fibrillary glomerulonephritis - Glomerulonephritis rapidly progressive - Membranoproliferative glomerulonephritis - Membranous glomerulonephritis - Mesangioproliferative glomerulonephritis - Tubulointerstitial nephritis and uveitis syndrome |  |
| Skin and subcutaneous tissue disorders |  |  |
| Alopecia areata |  |  |
| Autoimmune / immune-mediated blistering dermatoses | Including but not limited to:   - Bullous Dermatitis - Bullous Pemphigoid - Dermatitis herpetiformis - Epidermolysis bullosa acquisita (EBA) - Linear IgA-mediated bullous dermatosis (LABD), also known as Linear IgA disease - Pemphigus |  |
| Erythema multiforme |  |  |
| Erythema nodosum |  |  |
| Reactive granulomatous dermatitis | Including but not limited to   - Interstitial granulomatous dermatitis - Palisaded neutrophilic granulomatous dermatitis |  |
| Lichen planus | Includes liquen planopilaris |  |
| Localized Scleroderma (Morphoea) | Includes Eosinophilic fasciitis (also called Shulman syndrome) |  |
| Psoriasis |  |  |
| Pyoderma gangrenosum |  |  |
| Stevens-Johnson syndrome (SJS) | Including but not limited to:   - Toxic Epidermal Necrolysis (TEN) - SJS-TEN overlap |  |
| Sweet’s syndrome | Includes Acute febrile neutrophilic dermatosis |  |
| Vitiligo |  |  |
| Vasculitis |  |  |
| Large vessels vasculitis | Including but not limited to:   - Arteritic anterior ischemic optic neuropathy (AAION or arteritic AION) - Giant cell arteritis (also called temporal arteritis) - Takayasu's arteritis |  |
| Medium sized and/or small vessels vasculitis | Including but not limited to:   - Anti-neutrophil cytoplasmic antibody (ANCA) positive vasculitis (type unspecified) - Behcet's syndrome - Buerger’s disease (thromboangiitis obliterans) - Churg–Strauss syndrome (allergic granulomatous angiitis) - Erythema induratum (also known as nodular vasculitis) - Henoch-Schonlein purpura (also known as IgA vasculitis) - Microscopic polyangiitis - Necrotizing vasculitis - Polyarteritis nodosa - Single organ cutaneous vasculitis, including leukocytoclastic vasculitis, hypersensitivity vasculitis and acute hemorrhagic edema of infancy (AHEI) - Wegener's granulomatosis |  |
| Other (including multisystemic) |  |  |
| Anti-synthetase syndrome |  |  |
| Capillary leak syndrome | Frequently used related terms include: “systemic capillary leak syndrome (SCLS)” or “Clarkson's Syndrome” |  |
| Goodpasture syndrome | Frequently used related terms include: “pulmonary renal syndrome” and “anti-Glomerular Basement Membrane disease (anti-GBM disease)” |  |
| Immune-mediated enhancement of disease | Includes vaccine associated enhanced disease (VAED and VAERD). Frequently used related terms include “vaccine-mediated enhanced disease (VMED)”, “enhanced respiratory disease (ERD)”, “vaccine induced enhancement of infection”, “disease enhancement”, “immune enhancement”, and “antibody-dependent enhancement (ADE) |  |
| Immunoglobulin G4 related disease |  |  |
| Langerhans' cell histiocytosis |  |  |
| Multisystem inflammatory syndromes | Including but not limited to:   - Kawasaki’s disease - Multisystem inflammatory syndrome in adults (MIS-A) - Multisystem inflammatory syndrome in children (MIS-C) |  |
| Overlap syndrome |  |  |
| Raynaud’s phenomenon |  |  |
| Sarcoidosis | Includes Loefgren syndrome |  |
| Susac's syndrome |  |  |

Supplementary Table 3. Study objectives and endpoints

| **Objectives** | **Endpoints and estimands** |
| --- | --- |
| ***Primary*** |  |
| To demonstrate the non-inferiority of adjuvanted RSVPreF3 in terms of RSV-A neutralizing titers when co-administered with PCV20 compared to adjuvanted RSVPreF3 administered alone. | - RSV-A neutralizing titers expressed as between-groups geometric mean titer ratio, one month after the adjuvanted RSVPreF3 vaccine dose. |
| To demonstrate the non-inferiority of PCV20 when co-administered with adjuvanted RSVPreF3 compared to PCV20 administered alone. | - Opsonophagocytic titers for each of the pneumococcal vaccine serotypes expressed as between-groups geometric mean titer ratio, one month after the PCV20 dose |
| To demonstrate the non-inferiority of adjuvanted RSVPreF3 in terms of RSV-B neutralizing titers when co-administered with PCV20 compared to adjuvanted RSVPreF3 administered alone. | - RSV-B neutralizing titers expressed as between-groups geometric mean titer ratio, one month after the adjuvanted RSVPreF3 vaccine dose. |
| ***Secondary*** |  |
| To evaluate the humoral immune response to adjuvanted RSVPreF3 when co-administered with PCV20 or when administered separately. | - RSV-A neutralizing titers expressed as mean geometric increase over baseline one month after the adjuvanted RSVPreF3 dose. - RSV-B neutralizing titers expressed as mean geometric increase over baseline one month after the adjuvanted RSVPreF3 dose. |
| To evaluate the safety and reactogenicity following administration of adjuvanted RSVPreF3 and PCV20, co-administered or administered alone. | - Percentage of participants reporting each solicited event with onset within seven days after vaccine administration. - Percentage of participants reporting unsolicited adverse events within 30 days after vaccine administration. - Percentage of participants reporting serious adverse events after vaccine administration up to end-of-study (six months after last vaccination). - Percentage of participants reporting potential immune-mediated diseases after vaccine administration up to end-of-study (six months after last vaccination). |

Supplementary Table 4. Additional analysis of primary RSV immunogenicity endpoints

|  | **Per-protocol set** | **Exposed set** |
| --- | --- | --- |
| RSV-A |  |  |
| N / N’ | 382 / 512 | 511 / 535 |
| Adjusted GMT ratio (95%CI) | 1.06 (0.94–1.20) | 1.05 (0.94–1.17) |
| RSV-B |  |  |
| N / N’ | 382 / 511 | 512 / 534 |
| Adjusted GMT ratio (95%CI) | 0.99 (0.88–1.12) | 0.99 (0.89–1.10) |

Per-protocol set: set of all participants who complied with all protocol-specified requirements, and who had pre- and post-dose RSV immunogenicity results available; Exposed set: set of all enrolled participants who received at least one study intervention.

Adjusted GMT ratio, ratio of mean geometric titers (Control over Co-Ad); Co-Ad, participants who received a single dose of adjuvanted RSVPreF3 and a single dose of PCV20 on Day 1; Control, participants who received a single dose of PCV20 on Day 1 and a single dose of adjuvanted RSVPreF3 on Day 31; N, number of participants with available pre- and post-vaccination results in the control group; N’, number of participants with available pre- and post-vaccination results in the Co-Ad group; CI, confidence interval; RSV, respiratory syncytial virus;

Supplementary Table 5. Solicited administration site events within 7 days following each dose and overall (exposed set)

|  | **Co–Ad** | | **Control** | |
| --- | --- | --- | --- | --- |
|  | **n** | **% (95% CI)** | **n** | **% (95% CI)** |
| ***Pain*** |  |  |  |  |
| PCV20 dose |  |  |  |  |
| N | 537 |  | 546 |  |
| Any | 283 | 52.7 (48.4–57.0) | 208 | 38.1 (34.0–42.3) |
| Grade 3 | 7 | 1.3 (0.5–2.7) | 1 | 0.2 (0.0–1.0) |
| Adjuvanted RSVPreF3 dose |  |  |  |  |
| N | 537 |  | 510 |  |
| Any | 290 | 54.0 (49.7–58.3) | 225 | 44.1 (39.8–48.5) |
| Grade 3 | 4 | 0.7 (0.2–1.9) | 3 | 0.6 (0.1–1.7) |
| Per participant |  |  |  |  |
| N | 538 |  | 552 |  |
| Any | 336 | 62.5 (58.2–66.6) | 312 | 56.5 (52.3–60.7) |
| Grade 3 | 8 | 1.5 (0.6–2.9) | 4 | 0.7 (0.2–1.8) |
| Per dose |  |  |  |  |
| N | 1074 |  | 1056 |  |
| Any | 573 | 53.4 (50.3–56.4) | 433 | 41.0 (38.0–44.0) |
| Grade 3 | 11 | 1.0 (0.5–1.8) | 4 | 0.4 (0.1–1.0) |
| ***Erythema*** |  |  |  |  |
| PCV20 dose |  |  |  |  |
| N | 537 |  | 546 |  |
| Any | 24 | 4.5 (2.9–6.6) | 21 | 3.8 (2.4–5.8) |
| Grade 3 | 1 | 0.2 (0.0–1.0) | 2 | 0.4 (0.0–1.3) |
| Adjuvanted RSVPreF3 dose |  |  |  |  |
| N | 537 |  | 510 |  |
| Any | 54 | 10.1 (7.6–12.9) | 33 | 6.5 (4.5–9.0) |
| Grade 3 | 3 | 0.6 (0.1–1.6) | 0 | 0.0 (0.0–0.7) |
| Per participant |  |  |  |  |
| N | 538 |  | 552 |  |
| Any | 64 | 11.9 (9.3–14.9) | 47 | 8.5 (6.3–11.2) |
| Grade 3 | 4 | 0.7 (0.2–1.9) | 2 | 0.4 (0.0–1.3) |
| Per dose |  |  |  |  |
| N | 1074 |  | 1056 |  |
| Any | 78 | 7.3 (5.8–9.0) | 54 | 5.1 (3.9–6.6) |
| Grade 3 | 4 | 0.4 (0.1–1.0) | 2 | 0.2 (0.0–0.7) |
| ***Swelling*** |  |  |  |  |
| PCV20 dose |  |  |  |  |
| N | 537 |  | 546 |  |
| Any | 17 | 3.2 (1.9–5.0) | 17 | 3.1 (1.8–4.9) |
| Grade 3 | 2 | 0.4 (0.0–1.3) | 0 | 0.0 (0.0–0.7) |
| Adjuvanted RSVPreF3 dose |  |  |  |  |
| N | 537 |  | 510 |  |
| Any | 26 | 4.8 (3.2–7.0) | 21 | 4.1 (2.6–6.2) |
| Grade 3 | 0 | 0.0 (0.0–0.7) | 0 | 0.0 (0.0–0.7) |
| Per participant |  |  |  |  |
| N | 538 |  | 552 |  |
| Any | 38 | 7.1 (5.0–9.6) | 36 | 6.5 (4.6–8.9) |
| Grade 3 | 2 | 0.4 (0.0–1.3) | 0 | 0.0 (0.0–0.7) |
| Per dose |  |  |  |  |
| N | 1074 |  | 1056 |  |
| Any | 43 | 4.0 (2.9–5.4) | 38 | 3.6 (2.6–4.9) |
| Grade 3 | 2 | 0.2 (0.0–0.7) | 0 | 0.0 (0.0–0.3) |

Co-Ad, participants who received a single dose of adjuvanted RSVPreF3 and a single dose of PCV20 on Day 1; Control, participants who received a single dose of PCV20 on Day 1 and a single dose of adjuvanted RSVPreF3 on Day 31.

Note: In the Co-Ad group, solicited administration-site events were captured per arm within the single 7-day reporting period. In the Control group, these events were captured for the arm receiving the study vaccine during each of the two 7-day reporting periods. Participants who experienced a given reaction after each dose were counted only once in the ‘per participant’ category, while in the ‘per dose’ category, individuals may be counted more than once if the local reaction occurred after each dose.

CI, confidence interval; N, number of participants with completed eDiary card for each dose and per participant; n, number of events reported; PCV20, 20-valent pneumococcal conjugate vaccine; RSV, respiratory syncytial virus.

Supplementary Table 6. Summary of unsolicited AEs, SAEs and pIMDs (exposed set, without and with post‑hoc adjustment for receipt of concomitant non‑study vaccination)

|  | Exposed set | | | | | | Exposed set (post-hoc analysis) | | | | | |
| --- | --- | --- | --- | --- | --- | --- | --- | --- | --- | --- | --- | --- |
|  | Co-Ad  N=553 | | | Control  N=557 | | | Co-Ad  N=553 | | | Control  N=557 | | |
|  | occ | n | % (95% CI) | occ | n | % (95% CI) | occ* | n* | % (95% CI) | occ* | n* | % (95% CI) |
| Any unsolicited AE (within 30 days of any dose) | 86 | 60 | 10.8 (8.4–13.7) | 147 | 98 | 17.6 (14.5–21.0) | 73 | 56 | 10.1 (7.7–12.9) | 108 | 78 | 14.0 (11.2–17.2) |
| Any grade 3 | 4 | 4 | 0.7 (0.2–1.8) | 9 | 8 | 1.4 (0.6–2.8) | 3 | 3 | 0.5 (0.1–1.6) | 8 | 7 | 1.3 (0.5–2.6) |
| Any related | 21 | 17 | 3.1 (1.8–4.9) | 15 | 11 | 2.0 (1.0–3.5) | 18 | 14 | 2.5 (1.4–4.2) | 11 | 9 | 1.6 (0.7–3.0) |
| Any SAE (up to end of study) | 9 | 9 | 1.6 (0.7–3.1) | 19 | 14 | 2.5 (1.4–4.2) | 8 | 8 | 1.4 (0.6–2.8) | 17 | 13 | 2.3 (1.2–4.0) |
| Any pIMD (up to end of study) | 2 | 2 | 0.4 (0.0–1.3) | 1 | 1 | 0.2 (0.0–1.0) | 2 | 2 | 0.4 (0.0–1.3) | 1 | 1 | 0.2 (0.0–1.0) |
| Any fatal SAE (up to end of study) | 0 | 0 | 0.0 (0.0–0.7) | 2 | 2 | 0.4 (0.0–1.3) | 0 | 0 | 0.0 (0.0–0.7) | 2 | 2 | 0.4 (0.0–1.3) |

Co-Ad, participants who received a single dose of adjuvanted RSVPreF3 and a single dose of PCV20 on Day 1; Control, participants who received a single dose of PCV20 on Day 1 and a single dose of adjuvanted RSVPreF3 on Day 31.

AE, adverse event; CI, confidence interval; N, number of participants in the exposed set; n, number of participants reporting an event; occ, number of occurrences of the event; pIMD, potential immune-mediated disease, SAE, serious adverse event.

* Excludes participants who received SARS-CoV-2 +/- another non-study vaccination within 30 days of study vaccine administration and reported an unsolicited AE.
